# Management of Tonsillar Lipoma: Is Tonsillectomy Essential?

**DOI:** 10.1155/2014/451570

**Published:** 2014-02-04

**Authors:** Sohit P. Kanotra, Joel Davies

**Affiliations:** ^1^Department of Otolaryngology-Head and Neck Surgery, Louisiana State University, 533 Bolivar Street, New Orleans, LA 70112, USA; ^2^Faculty of Medicine, University of Toronto Medical School, Toronto, ON, Canada M5H 2J7

## Abstract

Tonsillar lipomas are rare benign tumors, with only a limited number of cases reported in the literature. Excision of the lipoma along with tonsillectomy has been proposed as the usual treatment option. We report a case of tonsillar lipoma which was managed by excision of the lesion without the need for a tonsillectomy. No recurrence was reported at a 2-year followup. A worldwide literature review was done to better define the clinical and histopathological features of these lesions. The authors propose that routine tonsillectomy is not required for these benign lesions and that simple excision of the stalk of the lipoma is sufficient.

## 1. Introduction

Benign tumors of the palatine tonsil are rare, usually presenting as polypoidal masses, and include papillomas, lymphangiomas, fibromas, and lipomas [1]. Although lipomas are the most common mesenchymal tumors of the body, only 15% of all lipomas occur in the head and neck region and are usually seen in the parotid gland, oral cavity, hypopharynx, retropharynx, and the larynx [1]. Lipomas of the tonsil are extremely rare with only a limited number of cases reported worldwide. We present a case of lipoma arising from the palatine tonsil which was managed with excision of the lesion without the need of a tonsillectomy. A literature review is done to discuss the varied clinical presentation of these rare tumors and to highlight the fact that, though benign, these lesions can have unusual and sometimes dangerous presentation.

## 2. Case Report

**Figure 1:**
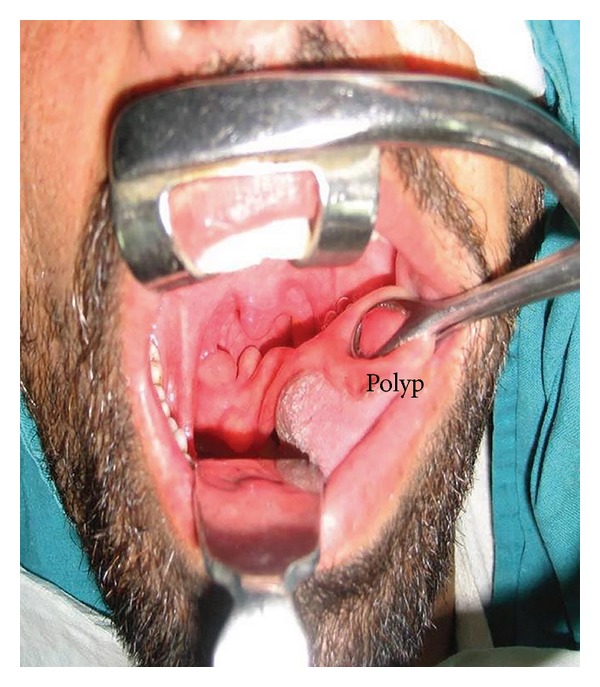
A large multilobulated polypoidal mass seen arising from the inferior pole of the right tonsillar fossa.

**Figure 2:**
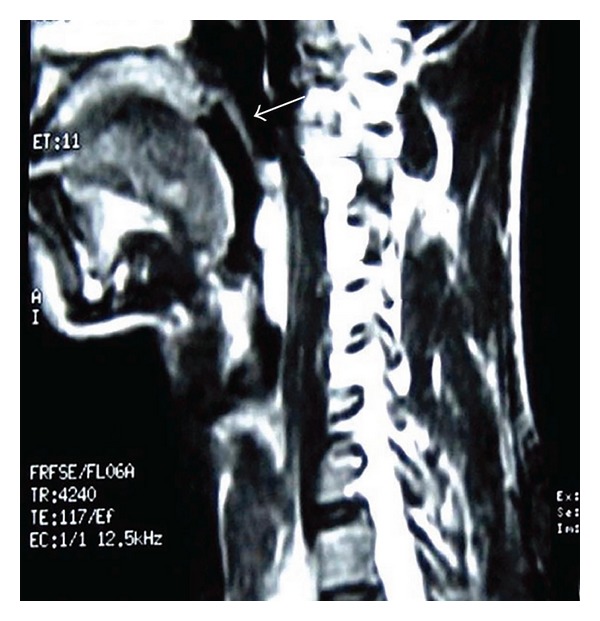
A T2-weighted MRI sagittal image showing the tonsillar polyp with the stalk arising from the inferior pole of the tonsil and extending posterior to the epiglottis and into the postcricoid region with partial occlusion of the glottis.

**Figure 3:**
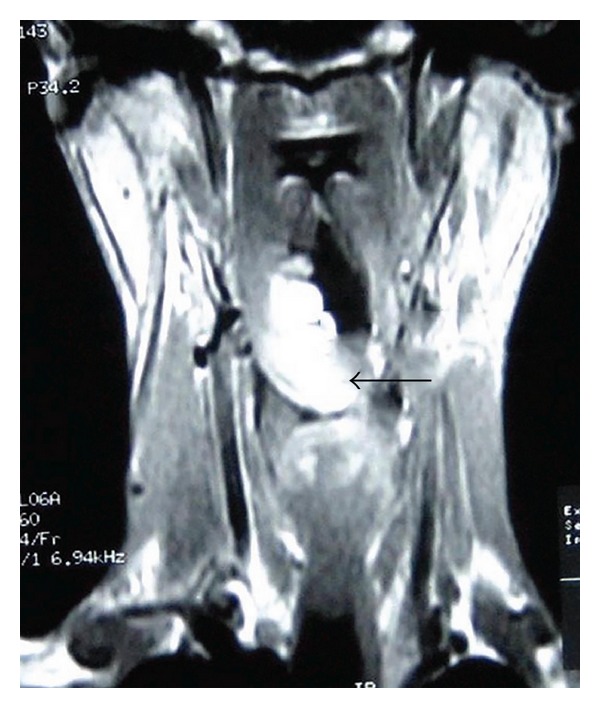
A T2-weighted MRI coronal image showing the hyperintense lesion.

**Figure 4:**
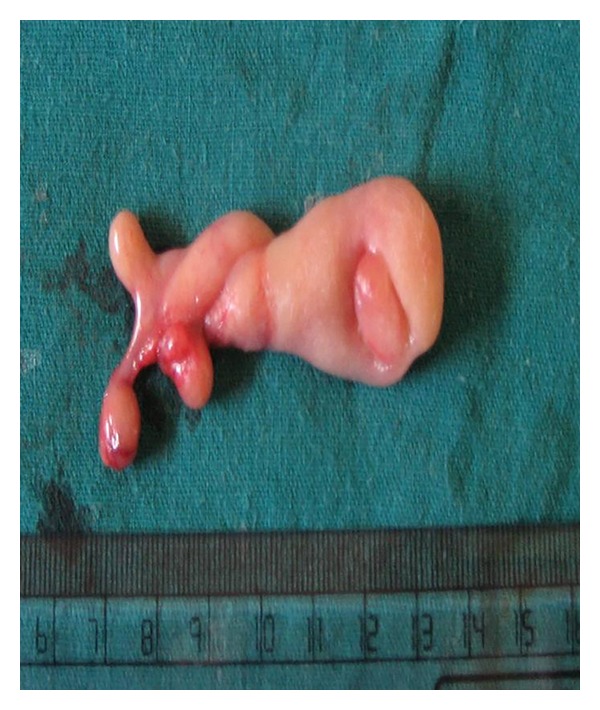
The excised tonsillar lipoma along with the stalk.

**Figure 5:**
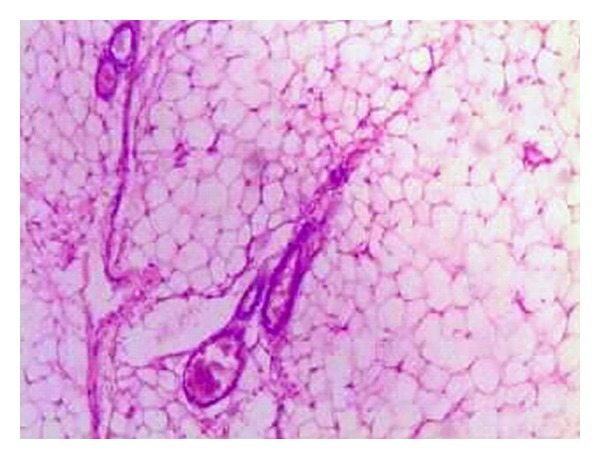
Histopathology of the excised specimen showing lobulated adipose tissue with scattered small vascular channels in the collagenous septa around the lobules (H&E, ×20).

A 28-year-old male presented with a one-year history of progressively increasing respiratory difficulty which was exacerbated in the left lateral decubitus position. The patient gave a history of multiple apneic spells at night. Upon initial examination of the oral cavity, no abnormality was observed. However, the patient, on making an effort, regurgitated a smooth surfaced polypoidal mass from the oropharynx. A large multilobulated polypoidal mass was seen arising from the inferior pole of the right tonsillar fossa and extending into the oropharynx (Figure 1). Laryngeal examination revealed a large smooth surfaced globular mass with intact mucosa obscuring the right pyriform fossa and partially occluding the airway. A MRI of the neck on T2-weighted sagittal image showed a hyperintense mass extending inferiorly from the lower pole of the right palatine tonsil into the oropharynx (Figures 2 and 3). A fat saturated image showed attenuation of the hyperintense lesion. The mass was excised under general anesthesia after clamping the base of the pedicle. The postoperative period was uneventful and a 2-year follow up of the patient revealed no recurrence of the tumour. Macroscopically the excised mass was 5 × 2 × 1 cm with a long stalk and a globular end (Figure 4). A microscopic examination revealed lobulated adipose tissue with scattered small vascular channels in the collagenous septa around the lobules (Figure 5).

## 3. Discussion

We present a case of tonsillar lipoma in a young adult which was managed with excision of the lesion without the need for a tonsillectomy and showed no recurrence at 2-year followup. The case highlights the fact that tonsillar lipomas can present with airway obstruction and that simple excision of the lesion without the need for tonsillectomy is sufficient for the management of these lesions.

Benign tumors of the palatine tonsil are rare and usually take the form of a polyp. The polypoidal lesions of the tonsil are named on the basis of the predominant tissue component found on histological examination as squamous papillomas, lymphangiectatic fibrous polyp, fibrovascular polyp, haemangioma, and lipoma. Of all these benign polypoidal lesions of the tonsil, squamous cell papillomas are the most common, followed by lymphangiomas [1].

Lipomas of the palatine tonsil are extremely rare lesions and only 23 cases have been reported so far (Table 1). A review of these cases reveals that tonsillar lipomas are predominantly seen in adults with a mean age of 48.5 ± 21.3 yrs and a range from 8 to 83 yrs, with no sex predilection. The development of symptomatic lesions usually takes time as they are exposed to constant swallowing and gravity leading to the formation of a pedunculated polypoidal lesion. Even though most of the time these are discovered incidentally [3, 5–7, 9, 11–14] tonsillar lipomas can present as cough [2], foreign body sensation [16, 19, 21, 22], voice change [15], airway obstruction [18], and even angina [22] or like in our case positional sleep apnea. Tonsillar lipomas have a potential to cause airway obstruction and hence should be managed in an expeditious manner. Tonsillar lipomas mostly arise from the body of the tonsil but can arise from the inferior pole as well as the peritonsillar space. Histologically, lipomas can be subclassified on the basis of other mesenchymal elements that form an intrinsic part of the tumor. The various variants include fibrolipomas, myxoid lipoma, angiolipoma, angiomyolipoma, spindle cell lipoma, chondroid lipoma, myolipoma, chondrolipoma, and osteolipoma with fibrolipomas being the most common.

Tonsillectomy with the excision of the lesion has been described as the most common treatment option for tonsillar lipomas, while surgical excision of the lipoma and stalk, without tonsillectomy, has rarely been described in the literature [9, 12]. The present case was managed with excision of the stalk without the need of tonsillectomy. Despite a more conservative management via surgical excision, no recurrence was observed at 2-year followup. Likewise, recurrence was not reported in two other cases where such a conservative management was employed. Adopting a more conservative approach of simple excision of the polyp along with the pedicle is sufficient for the management of these lesions without the need for a tonsillectomy, thus avoiding the postoperative morbidity associated with tonsillectomy, without increasing the likelihood of recurrence.

## 4. Conclusion

We present a case of tonsil lipoma presenting as sleep apnea and airway obstruction. Based on the twenty-three cases reported worldwide, it appears as though age and gender do not play a significant role in their development. While tonsillectomy remains the most frequently reported approach to removal of lipomas of the palatine tonsil, excision of the mass, without tonsillectomy, may represent a means of reducing risks of postoperative complications while maintaining low rates of recurrence.

## Figures and Tables

**Table 1:** Summary of tonsillar lipoma case reports including patient demographics, clinical features, and specimen information.

Author	Patient information			Specimen information			
	Age	Sex	Symptoms	Size (cm)	Side	Site	Histology
Theisen (1903) [2]	NA	NA	NA	NA	NA	NA	NA
Theisen (1903) [2]	8	F	Cough	0.7	R	Tonsil	Lipoma
New and Childrey (1931) [3]	NA	NA	Incidental	1.5 cm	R	Tonsillar fossa	Lipoma
Galetti (1959) [4]	NA	NA	NA	NA	NA	NA	Lipoma
Douglas (1961) [5]	55	M	Incidental	2 × 0.8	R	Tonsil	Fibrolipoma
Amendolea (1968) [6]	NA	M	Incidental	NA	NA	Tonsil	Fibrolipoma
Nizze (1974) [7]	NA	NA	Incidental	NA	L	Tonsil	Fibrolipoma
Krausen et al. (1986) [8]	NA	NA	NA	NA	R	Tonsil	Angiofibrolipoma
Begin and Frenkiel (1993) [9]	42	F	Incidental	NA	L	Tonsil	Lipoma
Tsunoda (1994) [10]	58	F	Oral mass	4 × 3 × 3	L	Peritonsillar Space	Lipoma
Benson-Mitchell et al. (1994) [11]	83	M	Incidental	6.5 × 2.5 × 1.4	L	Tonsil	Lipoma
Harada et al. (1995) [12]	44	F	Incidental	1.6 × 1.5 × 1.3	R	Tonsil	Lipoma
Gentile et al. (1996) [13]	72	M	Incidental	NA	NA	Tonsil	Spindle cell Lipoma
Sarma and Ramesh (1996) [14]	35	F	Incidental	1.2 × 1.0 × 0.8	L	Tonsil	Lipoma
M. R. Juvekar and R. V. Juvekar (2000) [15]	55	M	Dysphagia and inability to speak	13 × 1.5	R	Inferior pole	Myxoidn Lipoma
Halaas et al. (2001) [16]	65	M	Foreign body sensation	6.0 × 2.5 × 2.5	R	Inferior pole	Chondro lipoma
Band$\overset{´}{\text{e}}$ca et al. (2007) [17]	11	F	Incidental	NA	NA	Tonsil	Lipoma
Derek$\ddot{\text{o}}$y et al. (2007) [18]	63	F	Dyspnoea	3.6 × 3.2 × 2.2	R	Tonsil	Lipoma
Wang et al. (2007) [19]	46	F	Foreign body sensation	1 × 0.4 × 0.2	L	Tonsil	Lipoma
Martin et al. (2009) [20]	39	M	Cough	5 × 3 × 2	L	Tonsil	Fibrolipoma
Nandakumar et al. (2010) [21]	69	M	Foreign body sensation	3 × 1	L	Tonsil	Fibrolipoma
Sameh et al. (2012) [22]	62	F	Foreign body sensation and fever	1 × 0.5	R	Tonsil	Lipoma
Sameh et al. (2012) [22]	19	M	Angina	1.7 × 0.9	R	Tonsil	Lipoma

NA: not available.
